# Luminescence Properties of M(AlCl_4_)_2_:Yb^2+^ (M = Ca, Sr, Ba): Ideal Materials for the Investigation of Structure–Luminescence Relationships

**DOI:** 10.3390/ma17246193

**Published:** 2024-12-18

**Authors:** Julian Wilhelm Weiss, Natalie Kuhlmann, Claudia Wickleder

**Affiliations:** Inorganic Chemistry, University of Siegen, 57076 Siegen, Germany; weiss@chemie-bio.uni-siegen.de (J.W.W.); kuhlmann@chemie-bio.uni-siegen.de (N.K.)

**Keywords:** luminescence, divalent lanthanides, Yb^2+^

## Abstract

In this work, the chloride system M(AlCl_4_)_2_ (M = Ca, Sr, Ba) doped with Yb^2+^ is investigated in greater detail. The influence of the [AlCl_4_]^−^ ion on the position of the emission band of Yb^2+^ is investigated and the emission spectra are recorded. The emission spectra of the Yb^2+^-doped materials are characterized by broad 4f^13^5d^1^ (HS) ↔ 4f^14^ transitions with maxima in the range between 416 nm (Ca) and 421 nm (Ba) (24,061–23,738 cm^−1^), whereas the Ba compound features an additional 4f^13^5d^1^ (LS) ↔ 4f^14^ emission band at 397 nm (25,203 cm^−1^) at lower temperatures. The unusual reverse order of the positions of the emission maxima (Ba < Sr < Ca) most probably indicates an influence of the local symmetry on the location of the emission bands. Temperature-dependent emission and decay time measurements reveal strong thermal quenching processes, which gives additional insight into the mechanistic properties of the luminescence. An investigation of the decay times for all transitions reveals generally shorter values compared to values found in the literature, which was likely caused by the strong thermal quenching.

## 1. Introduction

Research on luminescent materials has been a focus for many years. In this context, research is largely focused on the divalent lanthanide Eu^2+^, which is the most chemically stable ion among the divalent lanthanides [1]. In comparison, the second most stable divalent lanthanide, Yb^2+^, has received significantly less attention, mostly due to its lower chemical stability. The optical transitions of Yb^2+^ are characterized by broad 4f^13^5d^1^ (HS/LS) → 4f^14^ spin-forbidden (HS) and spin-allowed (LS) emission bands [2], and due to the completely filled 4f^14^ orbital, no 4f ↔ 4f transitions can be observed. Moreover, semi-empirical crystal field calculations have been performed, since the excited 4f^13^5d^1^ configuration of Yb^2+^ exhibits only 140 microstates [3,4]. Similarly to the case for Eu^2+^, the energetic position of the excited states of Yb^2+^ is influenced by the crystal field induced according to the parameters of the corresponding crystallographic site of the host, and can be tuned throughout the visible spectrum. Halides and sulfates (MCl:Yb^2+^ (M = Na, K, Rb) [5], SrCl_2_:Yb^2+^ [6], BaSO_4_:Yb^2+^ [7]) show emissions in the UV to blue range, whereas green emission can be detected in certain nitride materials (α-SiAlON:Yb^2+^ [8]). Red emission is observed for sulfides and alkaline-earth nitride hosts (CaS:Yb^2+^ [9], SrSi_2_O_2_N_2_:Yb^2+^ [10]). Additionally, materials such as SrI_2_:Yb^2+^, CsBa_2_I_5_:Yb^2+^ and LaCl_3_:Yb^2+^ have been investigated for scintillator applications [11,12,13]. Furthermore, strong temperature quenching behavior can be detected in oxide materials [14]. However, only a few compound classes doped with Yb^2+^ have been investigated, but have nevertheless drawn attention to this field in recent years [15]. The structure–luminescence relationships of materials that feature complex halide anions and their effect on the luminescence properties of Yb^2+^ are scarcely understood. Recent studies revealed a strong influence of the vibrational energy on the occurrence of spin-allowed 4f^13^5d^1^ (LS) → 4f^14^ transitions, which is rarely observed in Yb^2+^-doped compounds [2]. Investigations of the Yb^2+^-doped halide perovskites CsCaX_3_:Yb^2+^ and CsSrX_3_:Yb^2+^ (X = Cl, Br, I) revealed an increase in the 4f^13^5d^1^ (LS) → 4f^14^ emission intensity compared to that of the 4f^13^5d^1^ (HS) → 4f^14^ emission as well as increasing the quenching temperatures of the LS emission bands in the order Cl < Br < I. The iodine compounds show exclusively spin-allowed LS transitions due to the low energy of the local vibrational modes [16]. In this context, the recently discussed compound class of alkaline-earth tetrachloroaluminates M(AlCl_4_)_2_ (M = Ca, Sr, Ba) featuring a [AlCl_4_]^−^ complex anion represent promising candidates for Yb^2+^ doping [17]. This pronounced anion provides better ionic bonding due to the Al^3+^ ion in the second coordination sphere, but at the same time features slightly lower energies for the local vibrational modes (*ћω* < 180 cm^−1^) compared to simple chlorides, e.g., CsCaCl_3_ (*ћω* = 200–240 cm^−1^) [16]. Although the alkaline-earth compounds Ca/Sr/Ba(AlCl_4_)_2_ feature identical stoichiometry, crystallization occurs in different crystallographic systems and provides distinct site symmetries for the alkaline-earth cationic site [18,19]. This material class can facilitate investigations into structure–property relationships, with regard to the impact of [AlCl_4_]^−^ as a complex anion on the luminescence of Yb^2+^. For the first time, an investigation of the photoluminescence characteristics of Yb^2+^-doped M(AlCl_4_)_2_ is carried out and discussed in this work.

## 2. Materials and Methods

The synthesis of the host material followed a procedure previously utilized in our group [17]. Since the procedure was modified to allow for the usage of Yb^2+^, the corresponding steps are discussed in greater detail. The sample preparation used CaCl_2_·2 H_2_O (Merck, Darmstadt, Germany, ≥99%), SrCl_2_·6 H_2_O (Riedel de-Häen, Seelze, Germany, 99%) and BaCl_2_·2 H_2_O (Merck, Darmstadt, Germany, >99.5%). The compounds had to be dried at 250 °C in a dynamic vacuum so that surface water and crystal water contents were removed. Furthermore, AlCl_3_ (Alfa Aesar, Haverhill, MA, USA, anhydrous, 99.985%) was used without any further purification steps. YbCl_2_ had to be prepared from Yb metal (Smart Elements, Vienna, Austria, 99.95%) and NH_4_Cl (Riedel de-Häen, Seelze, Germany, 99%) in a Schlenk setup using liquid ammonia (Messer SE & Co. KGaA, Bad Soden, Germany, 99.995%) [20]. For the synthesis of M(AlCl_4_)_2_:Yb^2+^ (M = Ca, Sr, Ba), a mixture of the dried binary starting materials, MCl_2_ (M = Ca, Sr, Ba) and AlCl_3_ (using a molar ratio of 1:2), as well as small amounts (0.1 mol%) of YbCl_2_, were melted at 400 °C in sealed graphitized quartz ampoules for 3 days, succeeded by a cooling step with a rate of 1 °C/h back to room temperature. A very low doping concentration was chosen to increase the resolution of the optical spectra and to prevent concentration quenching as well as structural influences often caused by higher doping concentrations. The usage of graphitized ampoules was crucial to prevent the oxidation of Yb^2+^ during the reaction. This synthesis, which was carried out in a Bridgman oven, yielded colorless, brittle crystals for Ca(AlCl_4_)_2_:Yb^2+^ and colorless, platelet-like, soft crystals for Sr/Ba(AlCl_4_)_2_:Yb^2+^. Because of the strong hygroscopic nature of the products and all the starting materials, every preparative step was carried out in a dry Ar atmosphere inside a glove box (MBraun, Garching, Germany). By using X-Ray powder diffraction, the phase purity of all Yb^2+^-doped samples discussed in this study could be confirmed by comparison with the respective theoretical patterns of the host material taken from the literature [18,19], as depicted in Figure S1. As the Yb^2+^ doping concentration of the investigated samples is quite low (0.1 mol%), no influence on the XRD pattern of the doped samples compared to the host material is expected. In the case of the unintentional doping of possible impurities, like unreacted starting materials, additional bands would be visible in the emission spectra. The absence of these can underline the phase purity of the investigated compounds.

The optical properties were analyzed with our equipment, which has also been used for previous studies [17]. Optical emission and excitation measurements were carried out using a Fluorolog3 FL3-22 spectrometer (Horiba Jobin Yvon, Oberursel, Germany), which was equipped with a 450 W Xenon arc lamp as a light source. The spectrometer features double Czerny–Turner monochromators, which allow for a spectral resolution down to 0.05 nm, and a photomultiplier tube, R928P (Hamamatsu Photonics, Herrschig am Ammersee, Germany), was used for signal detection. Temperature-dependent emission and excitation measurements down to 10 K were carried out inside a closed-cycle Helium cryostat (Advanced Research Systems, Inc., Macungie, PA, USA). Measurements above room temperature were carried out inside a home-built oven connected via wave guides to the spectrometer for signal detection. All emission spectra that were recorded were corrected for the sensitivity of the photomultiplier, and all excitation spectra were corrected for the background spectra of the lamp as part of the measurement process. Temperature-dependent decay time measurements in the region of ns to μs were conducted with a frequency-shifted Nd:YAG laser (Quanta Ray Indi 40, Spectra Physics, Darmstadt, Germany) with a repetition rate of 10 Hz. Selection of the desired emission wavelength was achieved by using a single monochromator (Horiba Jobin Yvon, Oberursel, Germany) with a resolution down to 5 nm. A photomultiplier attached to an oscilloscope (RTM 2052, 500 MHz 5G Sa/s, Rohde & Schwarz, Munich, Germany) was used for decay signal detection.

## 3. Results and Discussion

### 3.1. Description of the Crystal Structures of M(AlCl_4_)_2_ (M = Ca, Sr, Ba)

The samples presented in this work utilize host materials previously investigated with the divalent lanthanide Eu^2+^ [17]. The crystallographic features of these host materials are important for the interpretation of the optical properties. They feature exactly one eightfold coordinated M^2+^ site, which is subsequently occupied by Yb^2+^ ions during the doping process. The rest of the crystal structures consist of tetrahedral complex [AlCl_4_]^−^ anions. With regard to symmetry, the M^2+^ cationic sites of the host materials feature S_4_ (Ca), C_1_ (Sr) and C_2_ (Ba) symmetry [18,19]. Considering the ionic radii of the aforementioned M^2+^ ions in the eightfold coordination (Ca^2+^ = 112 pm, Sr^2+^ = 126 pm, Ba^2+^ = 142 pm), the Yb^2+^ ions (114 pm) match in size; therefore, successful doping must be assumed [21]. The M–Cl distances, *d*, increase with an increasing M^2+^ size (*d*(Ca-Cl) = 2.961 Å, *d*(Sr-Cl) = 3.046 Å, *d*(Ba-Cl) = 3.189 Å) [18,19].

### 3.2. Investigation of the Photoluminescence Properties of M(AlCl_4_)_2_:Yb^2+^

**Figure 1:**
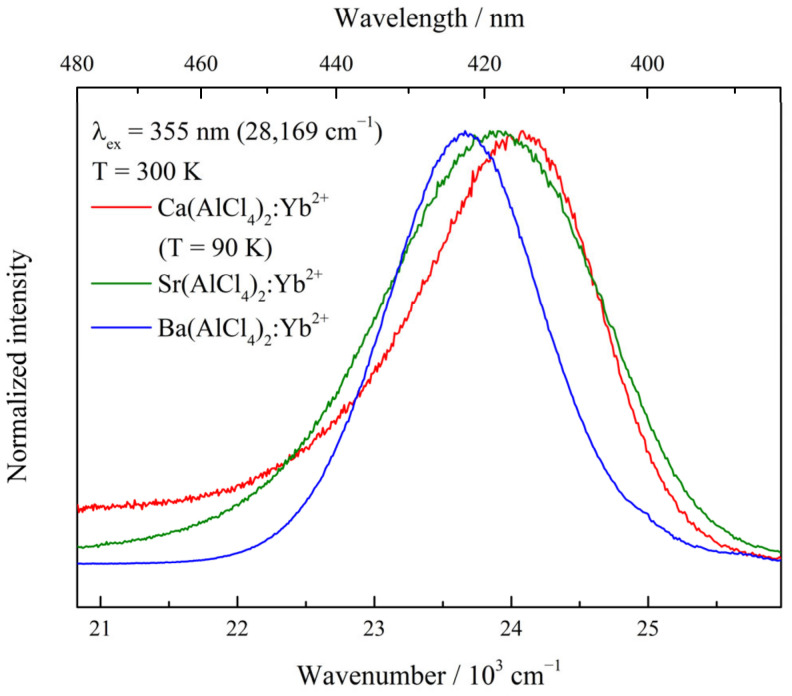
Emission spectra of M(AlCl_4_)_2_:0.1%Yb^2+^ recorded at 300 K with an excitation energy of λ_ex_ = 355 nm (28,169 cm^−1^). The emission spectrum of the Ca compound is depicted at T = 90 K.

All investigated materials show bright, violet to blue emission when irradiated with UV light, similar to the previously investigated Eu^2+^-doped samples [17]. Emission and excitation spectra were recorded at 300 K as well at 10 K for all samples. All results of the optical measurements, such as the position of the emission maxima, the determined Stokes shifts, ΔS, and information on the full width at half-maximum Γ (FWHM) of the emission bands are compiled in Table 1. In Figure 1, emission spectra of M(AlCl_4_)_2_:Yb^2+^ recorded at T = 300 K with an excitation wavelength of 355 nm (28,169 cm^−1^) are shown. An exception is Ca(AlCl_4_)_2_:Yb^2+^, which shows strong luminescence solely below 90 K. At elevated temperatures, strong thermal quenching is observed. Therefore, the emission spectrum of Ca(AlCl_4_)_2_:Yb^2+^ is shown for 90 K instead of 300 K.

**Figure 2:**
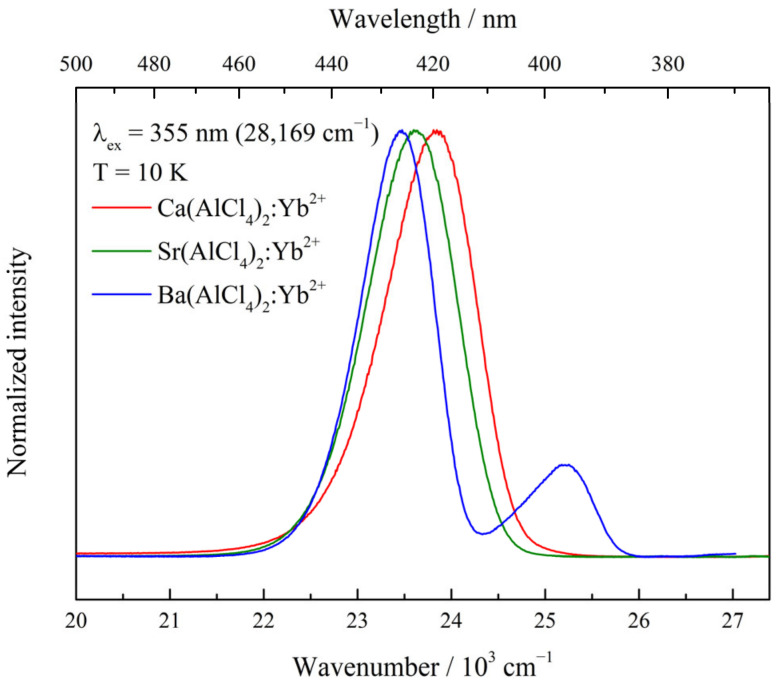
Emission spectra of M(AlCl_4_)_2_:0.1%Yb^2+^ recorded at 10 K with an excitation energy of λ_ex_ = 355 nm (28,169 cm^−1^).

All materials feature broad, spin-forbidden 4f^13^5d^1^ (HS) → 4f^14^ emission bands at higher temperatures. The respective emission maxima shift from 416 nm (24,061 cm^−1^, Ca, 90 K) to 419 nm (23,847 cm^−1^, Sr, 300 K) and, finally, to 421 nm (23,738 cm^−1^, Ba, 300 K), with FWHM values of Γ = 1551 cm^−1^ (Ca), 1869 cm^−1^ (Sr) and 1470 cm^−1^ (Ba), respectively. Upon cooling of the samples to 10 K, the position of the emission maxima changes to 420 nm (23,816 cm^−1^), 423 nm (23,614 cm^−1^), and 426 nm (23,462 cm^−1^) for the Ca-, Sr- and Ba- doped samples, respectively, whereas the associated values for Γ are 1152 cm^−1^, 1125 cm^−1^ and 925 cm^−1^ (Figure 2, Table 1).

Additionally, for Ba(AlCl_4_)_2_:Yb^2+^, a spin-allowed 4f^13^5d^1^ (LS) → 4f^14^ emission band located at 397 nm (25,203 cm^−1^) with an FWHM value of Γ = 759 cm^−1^ is observed. This band is, however, only visible at temperatures lower than 90 K. For all materials, the emission bands of the low temperature spectra compared to those measured at higher temperatures are red-shifted by about 4 nm. A declining population of the higher vibrational levels of the excited states at low temperatures is likely the cause. The decreasing Γ values can also be attributed to this fact.

In this circumstance, two observations are quite unexpected for the emission spectra. First, only the Ba compound shows a spin-allowed 4f^13^5d^1^ (LS) → 4f^14^ emission band at lower temperatures. This transition cannot be detected for the other two samples, even though the corresponding 4f^13^5d^1^ (LS) level has to be present. A possible explanation could be the position of the energetic levels of Yb^2+^ inside the band gap of the host lattices [22]. In the case of M(AlCl_4_)_2_:Yb^2+^ (M = Ca, Sr), the energetic levels are likely very close to the conduction band of M(AlCl_4_)_2_, so much so that only the excited 4f^13^5d^1^ (HS) level lies slightly below it, whereas the excited 4f^13^5d^1^ (LS) level lies within the conduction band. As such, no emission from this level can be observed due to photoionization. For Ba(AlCl_4_)_2_:Yb^2+^, the excited 4f^13^5d^1^ (LS) level lies slightly below the conduction band, and a transition can, therefore, be observed at low temperatures. This transition is then strongly quenched upon heating as higher lying vibrational states are populated, and those most likely already lie inside the conduction band. Additional insights into this observation can be gained by the analysis of the thermal quenching behavior.

**Figure 3:**
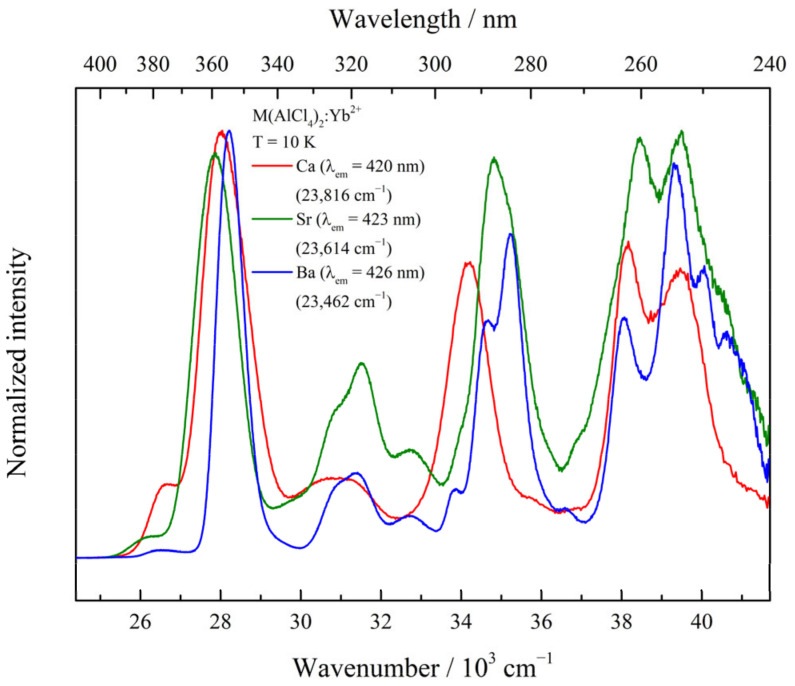
Excitation spectra of M(AlCl_4_)_2_:0.1%Yb^2+^ recorded at 10 K at the corresponding emission maxima (according to Table 1).

As for the second observation, in general, the crystal field splitting influencing the 5d levels will become larger with the M^2+^ ion’s decreasing radius. As a consequence, increasing crystal field splitting shifts the lowest 4f^13^5d^1^ level from higher to lower energies in the series Ba > Sr > Ca, as has been observed for Eu^2+^-doped compounds [17]. However, a contrary effect is observed in the present case. The emission bands shift from higher to lower energies following the series Ca > Sr > Ba. To discuss this behavior, the local M^2+^ site symmetry can be taken into consideration. It decreases from S_4_ (Ca) to C_1_ (Sr) and C_2_ (Ba). This will cause less degeneration of the excited 5d levels in the case of lower symmetries, resulting in larger splitting, which partially explains the observed behavior. Additional effects such as the covalency of the materials will have an influence on the position of the excited 5d states, and this might be another explanation for the position of the excited levels relative to the ground electronic state. The spectra of the excitation measurements of all materials performed at 10 K are depicted in Figure 3. Furthermore, separate emission and excitation spectra for each sample at 10 K are displayed in Figures S2–S4. An additional excitation spectrum was recorded for the 4f^13^5d^1^ (LS) → 4f^14^ emission and can be found in Figure S5. A high resolution of the spectra was achieved due to the low doping concentration, whereas the spectra recorded at higher temperatures were of a lower resolution (Figure S6). Several excitation bands in the range of 400–240 nm (25,000 cm^−1^–41,666 cm^−1^) can be observed for all samples and can be attributed to the different 4f^14^ → 4f^13^5d^1^ transitions, with positions depending on the respective M^2+^ site symmetry. Upon close inspection, similarities between the Sr and Ba compound are visible, compared to the Ca compound. This gives an indication that the observed order of the emission maxima is also influenced by the local site symmetry and that there may be a distorting effect due to the doping process, influencing the site symmetry conferred by the crystal structure.

A high resolution of the excitation spectra allows for a rather precise determination of the Stokes shift, where an increase can be observed with increasing M^2+^ radius (Table 1). The determined values are, in general, in agreement with those in the literature when compared to those for the halide perovskites CsCaCl_3_:Yb^2+^ and CsSrCl_3_:Yb^2+^ [2], for example.

### 3.3. Temperature-Dependent Emission Measurements

**Figure 4:**
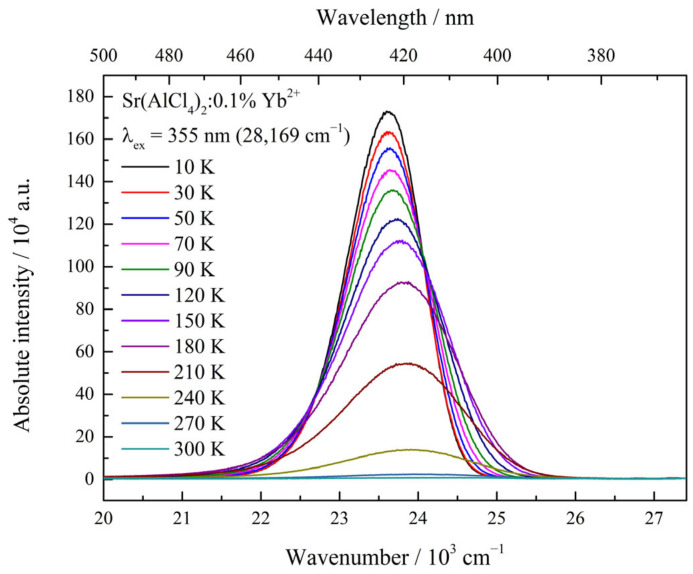
Temperature-dependent emission measurement of Sr(AlCl_4_)_2_:Yb^2+^ recorded with an excitation energy of λ_ex_ = 355 nm (28,169 cm^−1^). Thermal quenching can be observed.

**Figure 5:**
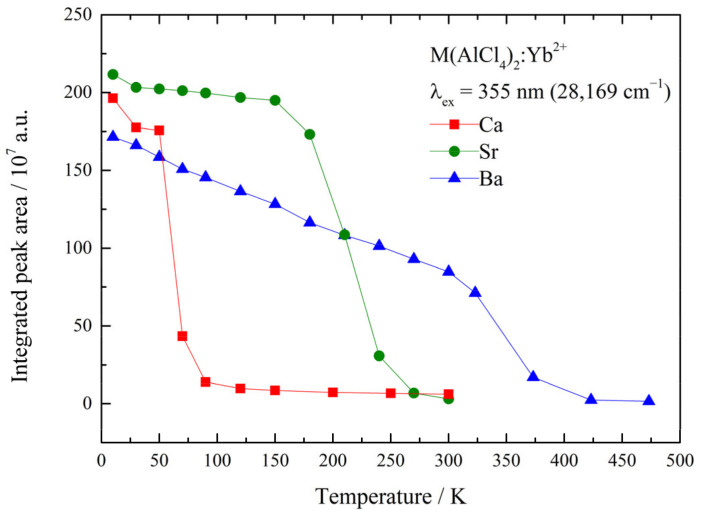
Temperature-dependent integrated peak areas of M(AlCl_4_)_2_:0.1%Yb^2+^. Thermal quenching is observed at different temperatures for all samples.

Furthermore, temperature-dependent luminescence measurements were performed using an excitation wavelength of λ_ex_ = 355 nm (28,169 cm^−1^). The results are shown as an example for the Sr compound in Figure 4, whereas the results for the Ca and Ba samples are presented in Figure S7–S9. An almost linear decrease in the position of the emission maxima was observed upon the heating of the samples, which was caused by the increasing population of higher vibrational levels of the excited states at increasing temperatures. A strong thermal quenching process was found for all samples, but the quenching temperature increased from Ca to Ba. The absolute integrated peak areas of the emission spectra are displayed in Figure 5. These observations are in agreement with the proposed positioning of the energetic levels of Yb^2+^ inside the band gap, as discussed previously alongside the findings in the literature [14].

### 3.4. Temperature-Dependent Decay Time Measurements

**Figure 6:**
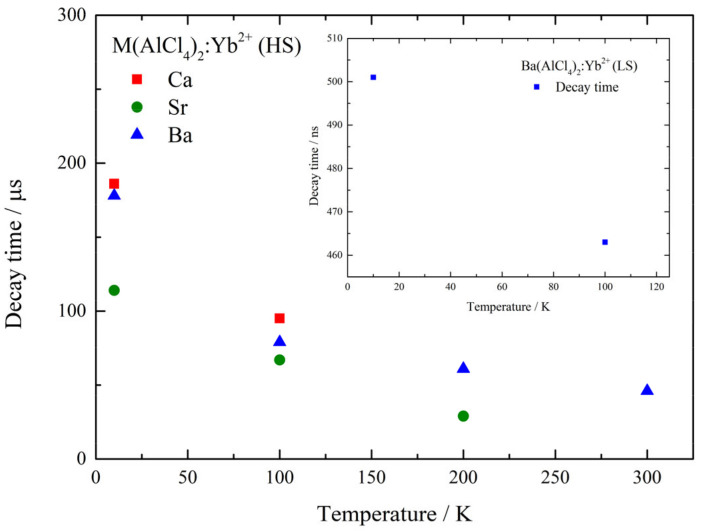
Temperature-dependent decay time measurements of M(AlCl_4_)_2_:0.1%Yb^2+^ at the corresponding emission maxima (Table 1), recorded with an excitation energy of λ_ex_ = 355 nm (28,169 cm^−1^).

To further investigate the nature of the observed transitions, temperature-dependent decay time measurements at the respective emission maxima were performed utilizing the previously used wavelength of λ_ex_ = 355 nm (28,169 cm^−1^) for excitation. The resulting decay curves are depicted in Figures S10–S12. To obtain the decay times shown in Table 2, (multi-)exponential decay functions are fitted to the decay signal, whereas the temperature-dependent behavior of the decay times for all compounds is shown in Figure 6. Due to insufficient emission intensity, decay times could not be determined for all transitions at every temperature step. In addition to the analysis of the transitions from the HS level, the temperature-dependent decay times of the 4f^13^5d^1^ (LS) → 4f^14^ emission of the Ba compound at λ_em_ = 397 nm (25,203 cm^−1^) were investigated (Figure S13), and the results are depicted in the insert of Figure 6. Spin-forbidden 4f^13^5d^1^ (HS) → 4f^14^ emissions usually feature longer decay times ranging from 1 to 10 ms, whereas the spin-allowed 4f^13^5d^1^ (LS) → 4f^14^ emissions have a shorter decay time of around 1 µs [23]. The observed decay times for the spin-forbidden HS transitions are slightly shorter than the corresponding values found in the literature. These findings indicate a quenching process which influences the decay times even at low temperatures. Similarly to the case during the thermal quenching observed in the emission measurements, the short decay times can also be explained by the close proximity of the excited 4f^13^5d^1^ levels of Yb^2+^ to the bottom of the conduction band of the host material causing quenching. Although the decay times follow the trend observed for the thermal quenching of the luminescence, different factors such as the density of crystal defects may also have influenced the observed decay times. The detected decay times for the spin-allowed LS emission in Ba(AlCl_4_)_2_:Yb^2+^ are also shorter than the corresponding values found in the literature for these kinds of transitions [23], which can be explained by the same reasoning given for the HS transitions.

## 4. Conclusions

In this work, the luminescence properties of M(AlCl_4_)_2_:Yb^2+^ (M = Ca, Sr, Ba) are presented for the first time. The emission measurements upon excitation with λ_ex_ = 355 nm (28,169 cm^−1^) show broad 4f^13^5d^1^ (HS) *→* 4f^14^ emission bands in the range of 416–421 nm (24,061–23,738 cm^−1^) for all investigated samples. Furthermore, Ba(AlCl_4_)_2_:Yb^2+^ shows an additional 4f^13^5d^1^ (LS) *→* 4f^14^ emission band at lower temperatures (397 nm, 25,203 cm^−1^). At 10 K, all emission maxima show a shift of about 4 nm to lower energies due to population of lower vibrational states. The reverse order of the emission maxima from lower (Ba) to higher (Ca) energy is observed for all temperatures, and this could be explained by the decreasing local crystallographic M^2+^ site symmetry, which led to the stronger splitting of the excited 5d levels. This is further supported by a comparison of the lowest-lying energetic level in the excitation spectra. All excitation spectra recorded at 10 K show well-resolved bands of Yb^2+^ and allow for the determination of the Stokes shift. Temperature-dependent emission measurements revealed strong temperature quenching for all samples. However, the exact temperatures varied and increased from Ca to Ba. This indicates the close proximity of the lowest excited levels of Yb^2+^ to the conduction band of the host materials. Moreover, this provides an explanation for the absence of the spin-allowed 4f^13^5d^1^ (LS) *→* 4f^14^ emission in Ca/Sr(AlCl_4_)_2_:Yb^2+^. The decay times determined for the spin-forbidden HS transitions are in the range of 30–200 µs, which is slightly shorter than comparable values found for other chloride compounds [23]. These decay times, which were influenced by quenching even at lower temperatures, can also be explained by the close proximity of the excited levels to the conduction band. The decay times for the spin-allowed LS emission of Ba(AlCl_4_)_2_:Yb^2+^ are in the range of 450–500 ns, which is slightly shorter than corresponding values found for other chloride compounds [23], and this can be explained by the same reasoning given above.

In general, these novel materials exhibit interesting properties and are ideal systems for the investigation of structure–luminescence relationships to understand the optical properties of divalent lanthanides.

## Figures and Tables

**Table 1:** Compilation of the optical properties of M(AlCl_4_)_2_:Yb^2+^ at selected temperatures (300 K/10 K). * Measured at T = 90 K.

	Ca(AlCl_4_)_2_	Sr(AlCl_4_)_2_	Ba(AlCl_4_)_2_
Emission maximum/nm	416*/420	419/423	421/426 (HS)
			397 (LS)
Stokes shift (ΔS)/cm^−1^	1342*/1524	1702/2015	2863/3020 (HS)
			928 (LS)
FWHM (Γ)/cm^−1^	1551*/1152	1869/1125	1470/925 (HS)
			759 (LS)

**Table 2:** Determined temperature-dependent decay times of M(AlCl_4_)_2_:Yb^2+^, at the corresponding emission maximum (Table 1).

Temperature/K	Ca(AlCl_4_)_2_/µs (HS)	Sr(AlCl_4_)_2_/µs (HS)	Ba(AlCl_4_)_2_ /µs (HS)/ns (LS)
10	186	114	178 (HS)/501 (LS)
100	95	67	79 (HS)/463 (LS)
200	-	29	65 (HS)/- (LS)
300	-	-	58 (HS)/- (LS)

## Data Availability

The original contributions presented in the study are included in the article/Supplementary Material. Further inquiries can be directed to the corresponding author.

## Supplementary Materials

The following supporting information can be downloaded at https://www.mdpi.com/article/10.3390/ma17246193/s1. Figure S1: Results of the XRD measurements of the M(AlCl_4_)_2_:0.1%Yb^2+^ (M = Ca, Sr, Ba) samples discussed in this study compared to the theoretical XRD patterns of the host material [18,19]. Reflections marked with a * are caused by the sample holder; Figure S2: Results of the emission and excitation measurements of Ca(AlCl_4_)_2_:0.1% Yb^2+^ at 10 K; Figure S3: Results of the emission and excitation measurements of Sr(AlCl_4_)_2_:0.1% Yb^2+^ at 10 K; Figure S4: Results of the emission and excitation measurements of Ba(AlCl_4_)_2_:0.1% Yb^2+^ at 10 K; Figure S5: Excitation spectra of the 4f^13^5d^1^ (LS) *→* 4f^14^ emission of Ba(AlCl_4_)_2_:0.1% Yb^2+^ at 10 K recorded at λ_em_ = 397 nm (LS, 25,203 cm^−1^); Figure S6: Excitation spectra of M(AlCl_4_)_2_:0.1% Yb^2+^ at T = 300 K (Ca: 90 K) recorded at their respective emission maximum; Figure S7: Results of the temperature-dependent emission measurements of Ca(AlCl_4_)_2_:Yb^2+^ recorded with an excitation energy of λ_ex_ = 355 nm (28,169 cm^−1^). A strong thermal quenching can be observed; Figure S8: Results of the temperature-dependent emission measurements of Ba(AlCl_4_)_2_:Yb^2+^ recorded with an excitation energy of λ_ex_ = 355 nm (28,169 cm^−1^). At lower temperatures, an additional emission band (LS) becomes visible; Figure S9: Results of the high temperature emission measurements of Ba(AlCl4)_2_:Yb^2+^ recorded with an excitation energy of λ_ex_ = 355 nm (28,169 cm^−1^). Thermal quenching of the HS transition occurs at around 350 K; Figure S10: Temperature-dependent decay time measurements of Ca(AlCl_4_)_2_:0.1% Yb^2+^ recorded with an excitation energy of λ_ex_ = 355 nm (28,169 cm^−1^); Figure S11: Temperature-dependent decay time measurements of Sr(AlCl_4_)_2_:0.1% Yb^2+^ recorded with an excitation energy of λ_ex_ = 355 nm (28,169 cm^−1^); Figure S12: Temperature-dependent decay time measurements of Ba(AlCl_4_)_2_:0.1% Yb^2+^ (HS) recorded with an excitation energy of λ_ex_ = 355 nm (28,169 cm^−1^); Figure S13: Temperature-dependent decay time measurements of Ba(AlCl_4_)_2_:0.1% Yb^2+^ (LS) recorded with an excitation energy of λ_ex_ = 355 nm (28,169 cm^−1^).
