# Supplementary material for: Luminescence Properties of M(AlCl4)2:Yb2+ (M = Ca, Sr, Ba): Ideal Materials for the Investigation of Structure–Luminescence Relationships
Source: Materials (Basel). 2024 Dec 18;17(24):6193. doi: 10.3390/ma17246193 (PMC11677979; doi:10.3390/ma17246193)
Supplement: Supplementary file 1 [file materials-17-06193-s001.zip › materials-3310876-supplementary.pdf]

Supplementary Materials

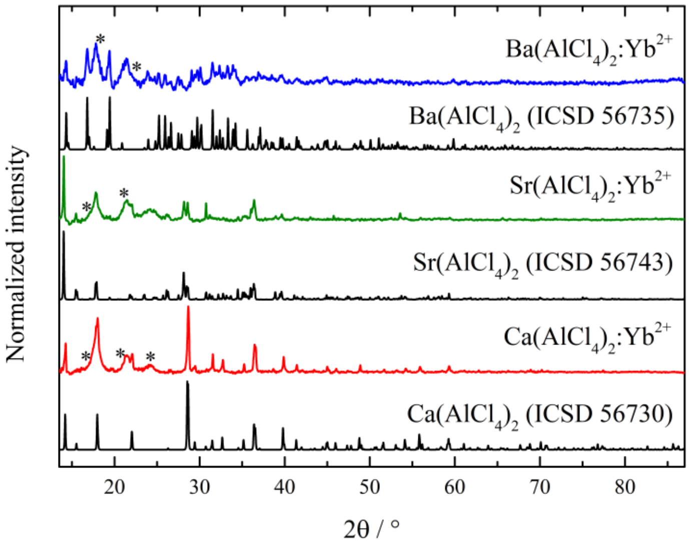

Figure S1. Results of the XRD measurements of the $M(AlCl_4)_2$:0.1%Yb^{2+} (M = Ca, Sr, Ba) samples discussed in this study compared to the theoretical XRD patterns of the host material [18,19]. Reflections marked with a * are caused by the sample holder.

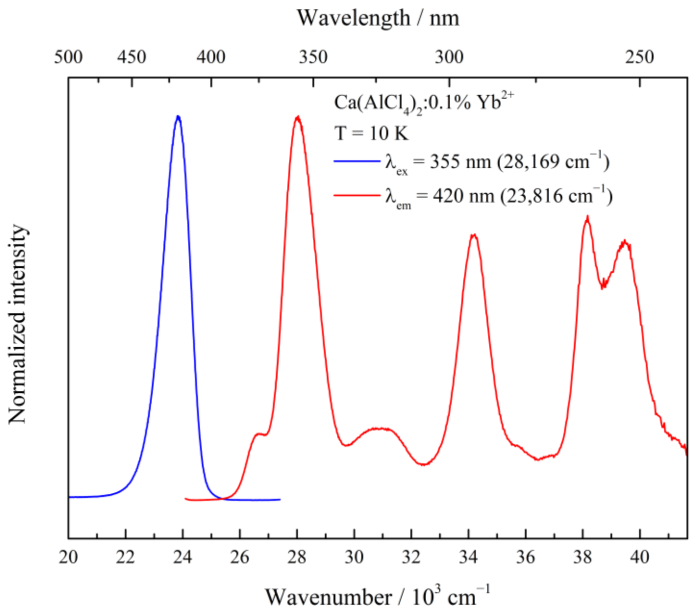

Figure S2. Results of the emission and excitation measurements of $Ca(AlCl_4)_2$:0.1% Yb^{2+} at 10 K.

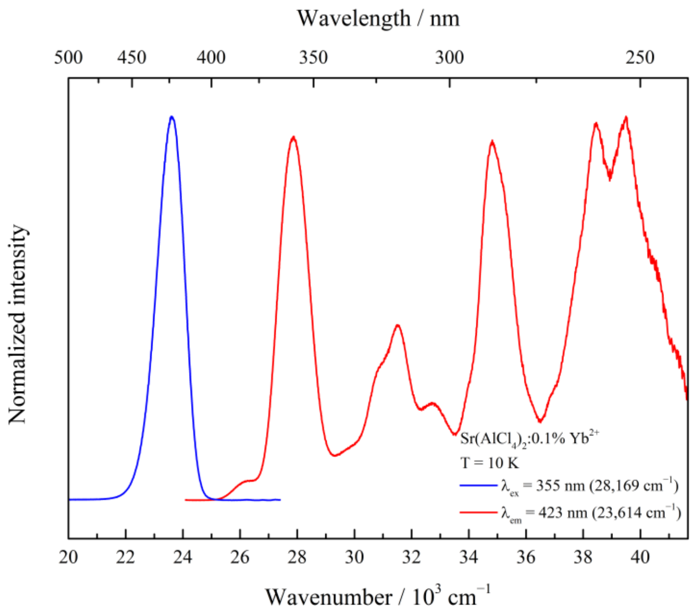

Figure S3. Results of the emission and excitation measurements of $Sr(AlCl_4)_2$:0.1% Yb^{2+} at 10 K.

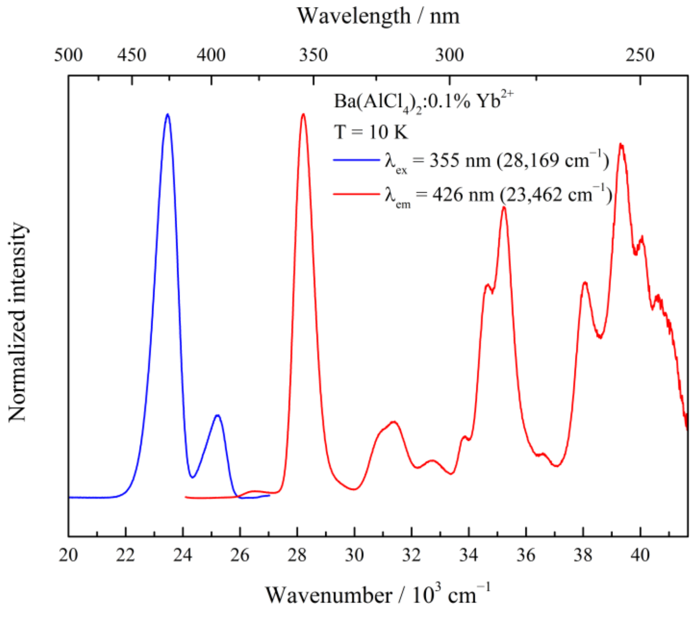

Figure S4. Results of the emission and excitation measurements of $Ba(AlCl_4)_2$:0.1% Yb^{2+} at 10 K.

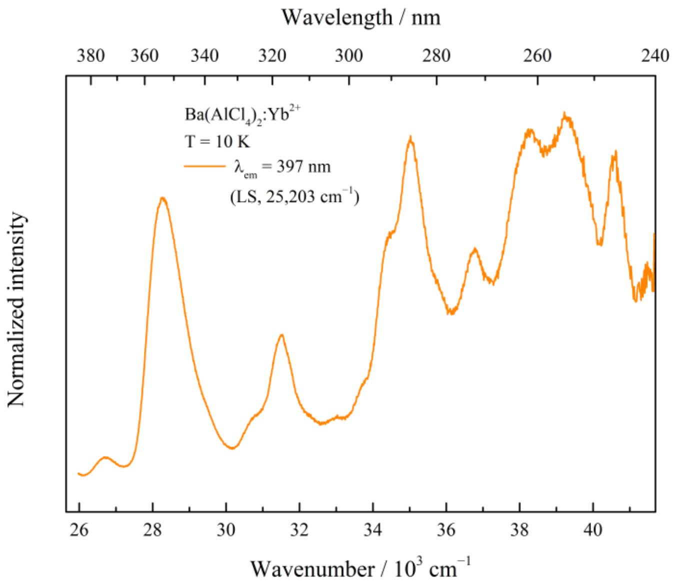

Figure S5. Excitation spectra of the $4f^{13}5d^1$ (LS) → $4f^{14}$ emission of $Ba(AlCl_4)_2$:0.1% Yb^{2+} at 10 K recorded at λ_{em} = 397 nm (LS, 25,203 cm^{-1}).

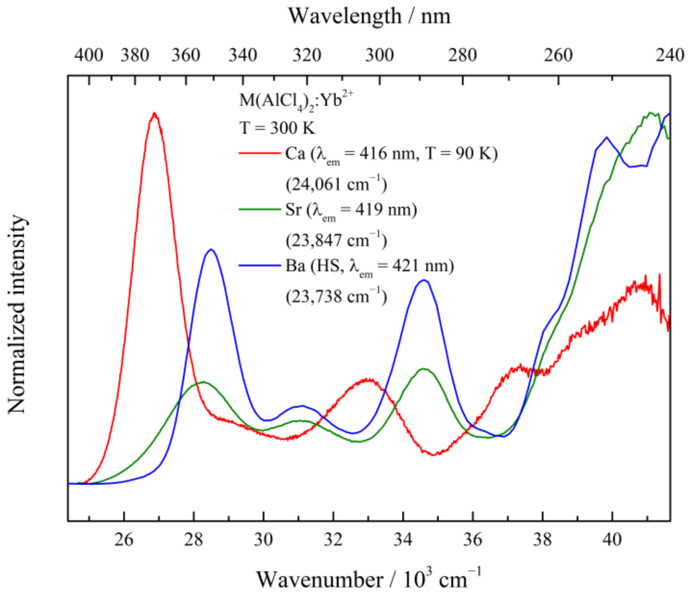

Figure S6. Excitation spectra of $M(AlCl_4)_2$:0.1% Yb^{2+} at T = 300 K (Ca: 90 K) recorded at their respective emission maximum.

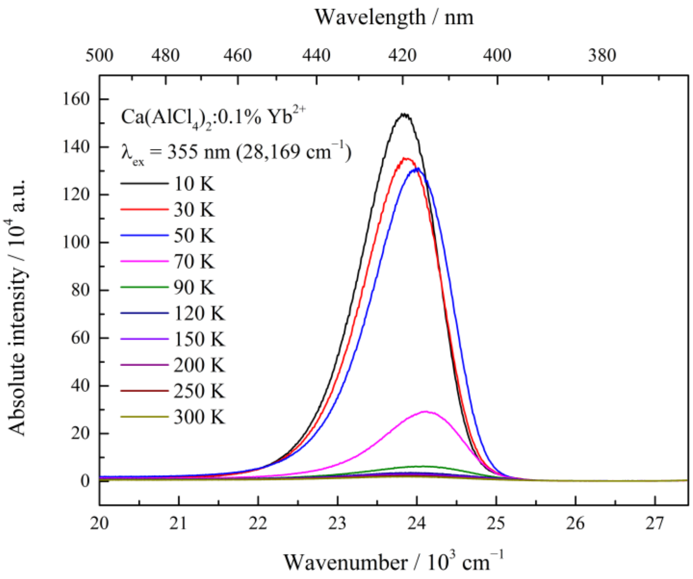

Figure S7. Results of the temperature-dependent emission measurements of $Ca(AlCl_4)_2:Yb^{2+}$ recorded with an excitation energy of λ_{ex} = 355 nm (28,169 cm^{-1}). A strong thermal quenching can be observed.

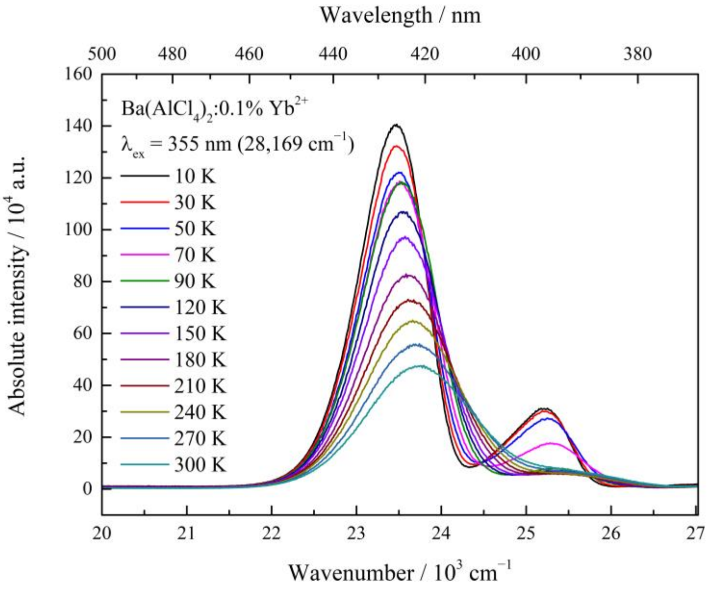

Figure S8. Results of the temperature-dependent emission measurements of $Ba(AlCl_4)_2:Yb^{2+}$ recorded with an excitation energy of λ_{ex} = 355 nm (28,169 cm^{-1}). At lower temperatures, an additional emission band (LS) becomes visible.

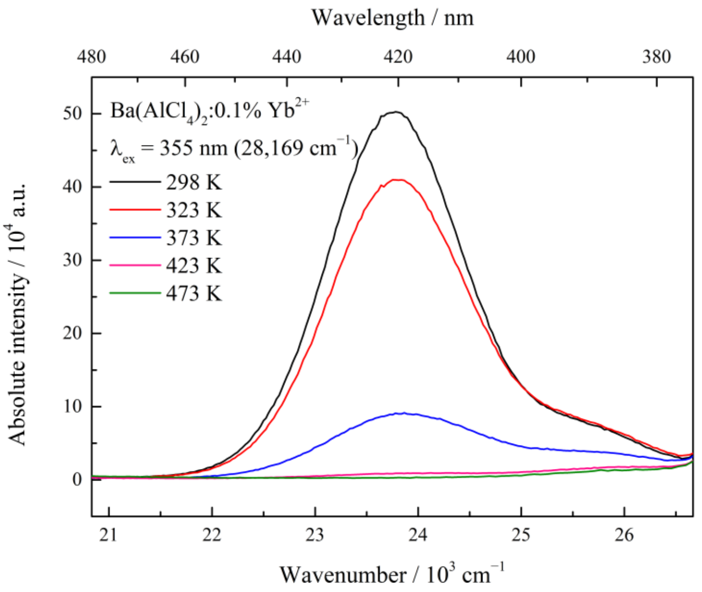

Figure S9. Results of the high temperature emission measurements of $Ba(AlCl4)_2:Yb^{2+}$ recorded with an excitation energy of λ_{ex} = 355 nm (28,169 cm^{-1}). Thermal quenching of the HS transition occurs at around 350 K.

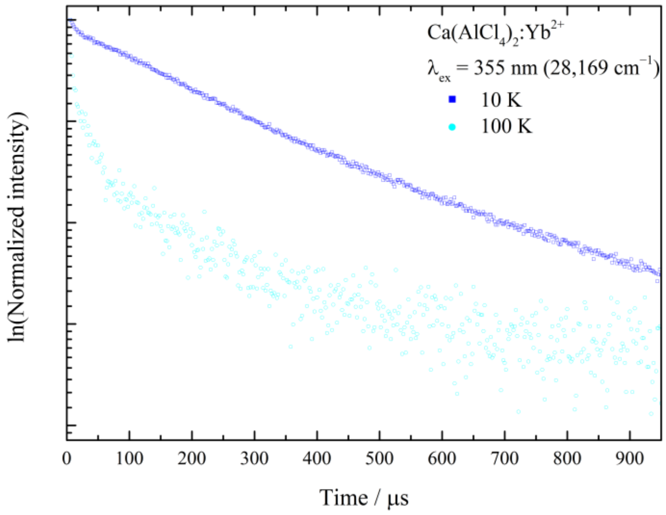

Figure S10. Temperature-dependent decay time measurements of $Ca(AlCl_4)_2$:0.1% Yb^{2+} recorded with an excitation energy of λ_{ex} = 355 nm (28,169 cm^{-1}).

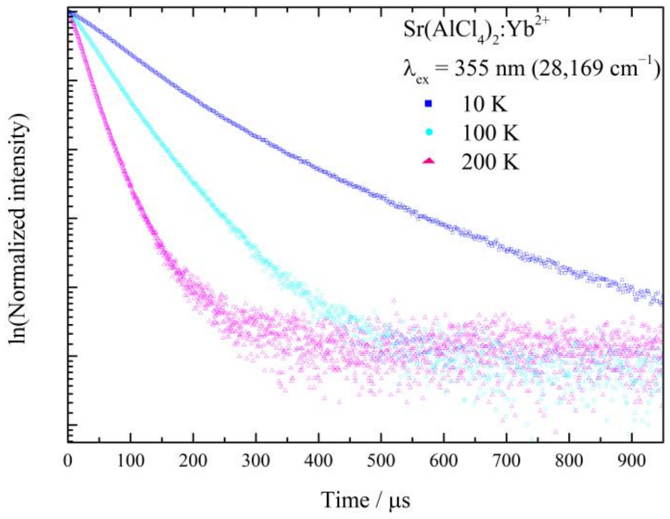

Figure S11. Temperature-dependent decay time measurements of $Sr(AlCl_4)_2$:0.1% Yb^{2+} recorded with an excitation energy of λ_{ex} = 355 nm (28,169 cm^{-1}).

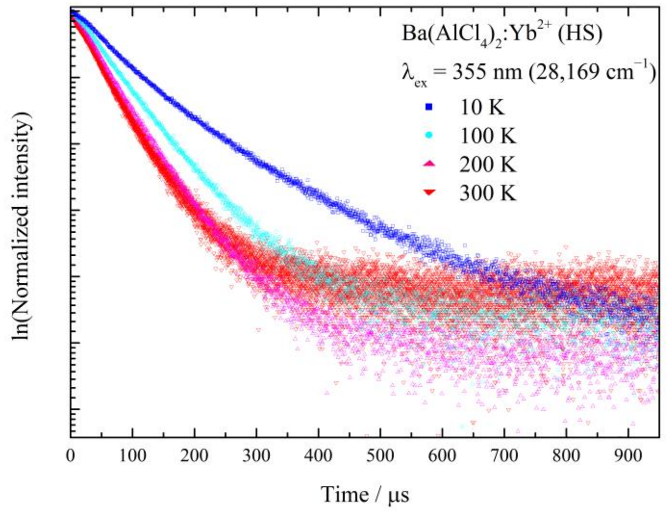

Figure S12. Temperature-dependent decay time measurements of $Ba(AlCl_4)_2$:0.1% Yb^{2+} (HS) recorded with an excitation energy of λ_{ex} = 355 nm (28,169 cm^{-1}).

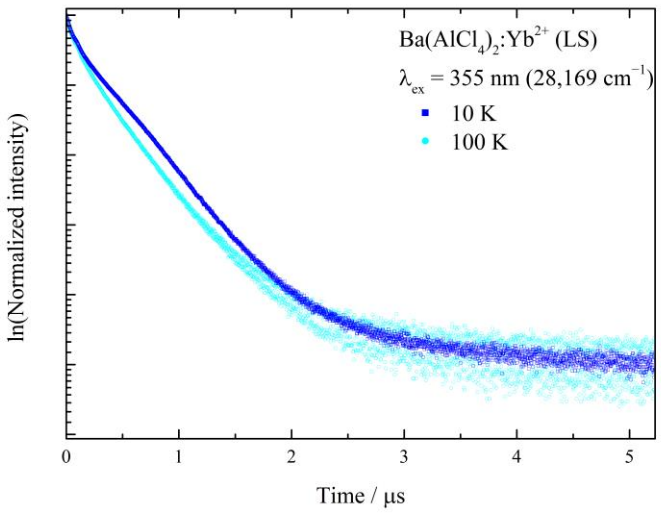

Figure S13. Temperature-dependent decay time measurements of $Ba(AlCl_4)_2$:0.1% Yb^{2+} (LS) recorded with an excitation energy of λ_{ex} = 355 nm (28,169 cm^{-1}).
